# Supplementary material for: Prevalence of underlying diseases in died cases of COVID-19: A systematic review and meta-analysis
Source: PLoS One. 2020 Oct 23;15(10):e0241265. doi: 10.1371/journal.pone.0241265 (PMC7584167; doi:10.1371/journal.pone.0241265)
Supplement: S1 File — (DOCX) [file pone.0241265.s001.docx]

| **Table S1:** Keywords and keyword combinations used to screen the PubMed, Scopus, and Web  of Science electronic databases |
| --- |
| (Covid-19) OR (((corona OR coronavirus)) AND death) OR (2019 novel coronavirus infection AND died) OR (COVID19 AND mortality) OR (coronavirus disease 2019 AND deceased) OR (coronavirus disease-19 AND fatality) OR  (2019-nCoV disease AND death) OR (2019 novel coronavirus disease AND mortality) OR (2019-nCoV infection and Death) |

| **TableS2. Quality assessment of included articles** | | | | |
| --- | --- | --- | --- | --- |
| Authors | **Selection** | **Comparability** | **Exposure/Outcome** | **Total Score** |
| Fan Yang, et a | ***** | * | *** | ********* |
| Qiurong Ruan, et al | ***** | ** | *** | ********** |
| Mark M. Alipio, et al | ***** | ** | *** | ********** |
| Jianfeng Xie, et al | ***** | * | ** | ******** |
| Wei-jie Guan, et al | ***** | ** | *** | ********** |
| Francesco Violi, et al | ***** | ** | *** | ********** |
| Amir Emami, et al | ***** | * | ** | ******** |
| Reza Shahriarirad, et al | ***** | ** | *** | ********** |
| Mohammad Nikpouraghadam, et al | ***** | * | *** | ********* |
| Yan Deng, et al | **** | ** | *** | ********* |
| Yongli Yan, et al | **** | ** | *** | ********* |
| Graziano Onder, et al | ***** | * | *** | ********* |
| Marcello Covino, et al | ***** | ** | *** | ********** |
| Xun Li1, et al | ***** | ** | *** | ********** |
| Mingli Yuan, et al | ***** | * | ** | ******** |
| Fei Zhou, et al | ***** | ** | *** | ********** |
| Mingli Yuan, et al | ***** | ** | *** | ********** |
| [Jianlei Cao, et al](javascript:;) | ***** | * | ** | ******** |
| Jianbo Tian, et al | **** | ** | *** | ********* |
| Lang Wang, et al | **** | ** | *** | ********* |
| Chaomin Wu, et al | ***** | * | ** | ******** |
| Rong-Hui Du, et al | ***** | * | *** | ********* |
| Bicheng Zhang, et al | ***** | * | *** | ********* |
| Kunyu Yang, et al | ***** | ** | *** | ********** |
| [Yingzhen Du, et al](https://pubmed.ncbi.nlm.nih.gov/?term=Du+Y&cauthor_id=32242738) | ***** | ** | *** | ********** |
| Chaomin Wu, et al | ***** | * | ** | ******** |
| CDC Korea | ***** | ** | *** | ********** |
| [Yifei Chen, et al](https://www.ncbi.nlm.nih.gov/pubmed/?term=Chen%20Y%5BAuthor%5D&cauthor=true&cauthor_uid=32373340) | ***** | ** | *** | ********** |
| Ya-Jun Sun, et al | ***** | * | ** | ******** |
| Junli Li, et al[[15](#_ENREF_15)] | ***** | ** | *** | ********** |
| Yiguang Chen, et al | ***** | * | *** | ********* |
| Lei Chen, et al | **** | ** | *** | ********* |

**SELECTION**

1) Representativeness of the Exposed Cohort

2) Selection of the Non-Exposed Cohort

3) Ascertainment of Exposure

4) Demonstration That Outcome of Interest Was Not Present at Start of Study

**COMPARABILITY**

1. Comparability of Cohorts on the Basis of the Design or Analysis

**OUTCOME**

1. Assessment of Outcome
2. Was Follow-Up Long Enough for Outcomes to Occur
3. 3) Adequacy of Follow Up
