# Supplementary material for: Prevalence of underlying diseases in died cases of COVID-19: A systematic review and meta-analysis
Source: PLoS One. 2020 Oct 23;15(10):e0241265. doi: 10.1371/journal.pone.0241265 (PMC7584167; doi:10.1371/journal.pone.0241265)
Supplement: S2 File — (DOCX) [file pone.0241265.s002.docx]

Figure S1. Funnel plot for meta-analysis of the prevalence of hypertensive patients in COVID-19 died cases.

Figure S2. Funnel plot for meta-analysis of the prevalence of diabetic patients in COVID-19 died cases.

Figure S3. Funnel plot for meta-analysis of the prevalence of cardiovascular disease in COVID-19 died cases.

Figure S4. Funnel plot for meta-analysis of the prevalence of kidney disease in COVID-19 died cases.

Figure S5. Funnel plot for meta-analysis of the prevalence of COPD in COVID-19 died cases.

Figure S6. Funnel plot for meta-analysis of the prevalence of malignancy in COVID-19 died cases.

Figure S7. Funnel plot for meta-analysis of the prevalence of liver disease in COVID-19 died cases.

Figure S8. Funnel plot for meta-analysis of the prevalence of lung disease in COVID-19 died cases.

Figure S9. Funnel plot for meta-analysis of the prevalence of cerebrovascular disease in COVID-19 died cases.
