# Supplementary material for: Prevalence of underlying diseases in died cases of COVID-19: A systematic review and meta-analysis
Source: PLoS One. 2020 Oct 23;15(10):e0241265. doi: 10.1371/journal.pone.0241265 (PMC7584167; doi:10.1371/journal.pone.0241265)
Supplement: Attachment — Submitted filename: Rebuttal Letter.docx [file pone.0241265.s004.docx]

Thanks the reviewer for positive insight to our article.

Here I provide the point by point response to the comments.

**Editor Comments**

**Comments:** please upload your study’s minimal underlying data set as either Supporting Information files or to a stable, public repository and include the relevant URLs, DOIs, or accession numbers within your revised cover letter.

**Answer:** the current article is a systematic review and meta-analysis study. It does not have any original data. By this way I provide the table of included studies as a attached file with name Supporting Information.

**Comments:** Please amend either the title on the online submission form (via Edit Submission) or the title in the manuscript so that they are identical.

**Answer**: the title is “Prevalence of Underlying Diseases in died cases of COVID-19: a Systematic Review and Meta-Analysis”. It is identical in submission form and article file.

**Comments:** Please remove your figures from within your manuscript file, leaving only the individual TIFF/EPS image files, uploaded separately.  These will be automatically included in the reviewers’ PDF.

**Answer**: All the figures removed from the article and submitted separately.

**Comments:** Please include captions for your Supporting Information files at the end of your manuscript, and update any in-text citations to match accordingly.

Answer: Thanks for your comment. I add the explanation of my supplementary file.

Supplementary file1: Search terms and quality assessment tables

Supplementary file2: Funnel plot for publication bias assessment

**Reviewers' comments:**

**Comments:** The paper is very interesting and informative. I only suggest to deepen a little bit the issue of the relationship with cardiovascular disease in the discussion section. For example discuss and cite the following recent article on this topic: Ielapi N, et al. Cardiovascular disease as a biomarker for an increased risk of COVID-19 infection and related poor prognosis. Biomark Med. 2020 Jun;14(9):713-716.

Answer:Thanks for your suggestion. I add more explanation about cardiovascular disease in the discussion section and add the reference.
